# The intricate interplay between ferroptosis and efferocytosis in cancer: unraveling novel insights and therapeutic opportunities

**DOI:** 10.3389/fonc.2024.1424218

**Published:** 2024-10-31

**Authors:** Ali Ahmadizad Firouzjaei, Samira Mohammadi-Yeganeh

**Affiliations:** Shahid Beheshti University of Medical Sciences, Tehran, Iran

**Keywords:** cancer, efferocytosis, ferroptosis, inflammation, tissue homeostasis

## Abstract

The complex interplay between ferroptosis and efferocytosis in cancer has attracted significant interest recently. Efferocytosis, the process of eliminating apoptotic cells, is essential for preserving tissue homeostasis and reducing inflammation. However, dysregulation of efferocytosis can have profound effects on cancer. Apoptotic cells accumulate because of impaired efferocytosis, which triggers chronic inflammation and the release of pro-inflammatory chemicals. Surprisingly, accumulating evidence suggests that dysregulation of ferroptosis- a form of controlled cell death characterized by lipid peroxidation and the buildup iron-dependent reactive oxygen species (ROS)-can influence efferocytic activities within the tumor microenvironment. Dysfunctional iron metabolism and increased lipid peroxidation, are associated with ferroptosis, resulting in inadequate apoptotic cell clearance. Conversely, apoptotic cells can activate ferroptotic pathways, increasing oxidative stress and inducing cell death in cancer cells. This reciprocal interaction emphasizes the complex relationship between efferocytosis and ferroptosis in cancer biology. Understanding and managing the delicate balance between cell clearance and cell death pathways holds significant therapeutic potential in cancer treatment. Targeting the efferocytosis and ferroptosis pathways may offer new opportunities for improving tumor clearance, reducing inflammation, and sensitizing cancer cells to therapeutic interventions. Further research into the interaction between efferocytosis and ferroptosis in cancer will provide valuable insights for the development of novel therapies aimed at restoring tissue homeostasis and improving patient outcomes.

## Higlights

- The dysregulation of efferocytosis in cancer leads to the accumulation of apoptotic cells, resulting in chronic inflammation and the release of pro-inflammatory chemicals.
- Enhanced lipid peroxidation linked to ferroptosis and dysfunctional iron metabolism can impede effective efferocytosis, resulting in inadequate clearance of apoptotic cells.
- Ferroptosis pathways can be triggered by the presence of apoptotic cells, which increase oxidative stress and kill cancer cells.
- The reciprocal interaction between efferocytosis and ferroptosis highlights the complex interplay of these processes in cancer biology.
- Significant therapeutic potential exists for the treatment of cancer if the delicate balance between cell death and clearance pathways is understood and managed.

## 1 Introduction

In recent years, the intricate relationship between cell clearance and cell death has emerged as a fascinating area of research in cancer biology. Two distinct cellular processes, namely efferocytosis and ferroptosis, have gained considerable attention due to their pivotal roles in maintaining tissue homeostasis and influencing tumor progression. Efferocytosis refers to the clearance of apoptotic cells by phagocytes, while ferroptosis is a regulated form of cell death characterized by lipid peroxidation dependent on iron (1, 2).

Cancer development and progression are often accompanied by an imbalance in these processes, leading to disrupted cell clearance and altered cell death mechanisms (3–5). It is now evident that the interplay between efferocytosis and ferroptosis plays a critical role in shaping the tumor microenvironment and influencing cancer outcomes. Understanding the intricate crosstalk between these two processes is thus essential for uncovering novel therapeutic strategies and improving patient outcomes (6–9).

This article aims to provide a comprehensive overview of the interplay between efferocytosis and ferroptosis in cancer, highlighting the delicate balance between cell clearance and cell death. We will explore the underlying molecular mechanisms governing these processes and discuss their implications in cancer initiation, progression, and therapy resistance. Additionally, we will examine emerging therapeutic approaches targeting efferocytosis and ferroptosis as potential strategies for cancer treatment.

By elucidating the complex relationship between efferocytosis and ferroptosis, this article will contribute to a deeper understanding of the molecular mechanisms underlying cancer biology. Furthermore, it will shed light on novel therapeutic opportunities for modulating cell clearance and cell death pathways, potentially opening new avenues for personalized cancer therapies.

## 2 Phagocyte recognition, engulfment, and degradation of apoptotic cells

**Figure 1:**
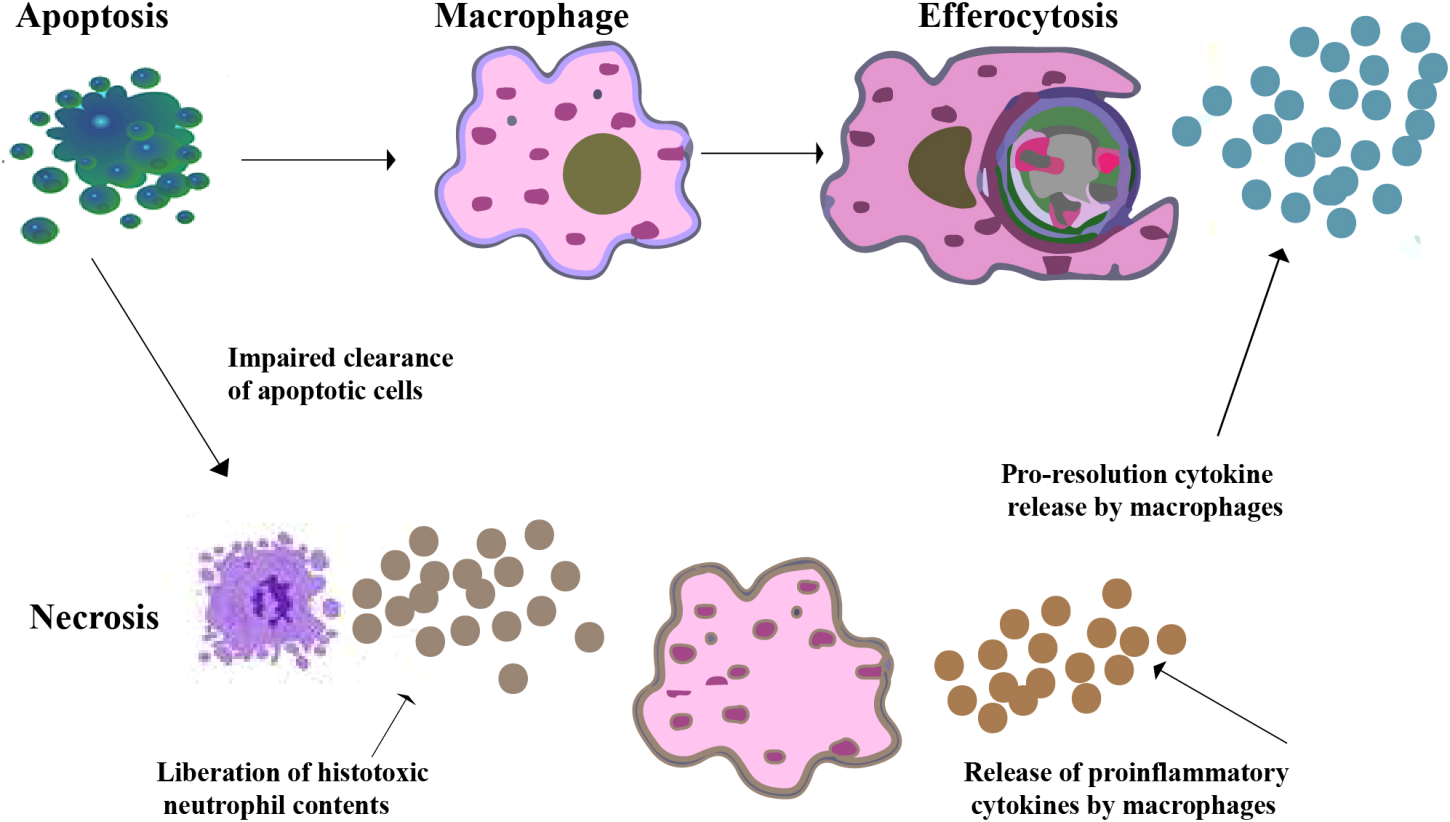
Efferocytosis by macrophages. A macrophage recognizes and phagocytoses an apoptotic neutrophil at an inflammatory site. Pro-resolution cytokines are secreted by macrophages while pro-inflammatory cytokines are downregulated as a result of their contact with apoptotic cells. Apoptotic neutrophils may undergo secondary necrosis if efferocytosis is impaired, which leading to the release of histotoxic substances, cause tissue damage, increased pro-inflammatory signals, and chronic inflammation.

Daily, approximately 0.4% of the estimated 37.2 trillion cells in the adult human body undergo programmed cell death (10). However, even in tissues characterized by high rate of cellular turnover, apoptotic cells (ACs) are remarkably rare, highlighting the extraordinary efficiency and vast capacity of AC clearance. This process, known as “efferocytosis”, is crucial for maintaining tissue homeostasis under normal physiological conditions and for restoring equilibrium after disease (11, 12). During the initial stage of efferocytosis, phagocytes recognize apoptotic cells by detecting “find-me” signals released as cells undergo their final stages of death. These signals consist of a wide range of lipids, proteins, peptides, and complex structures (13, 14). Additionally, apoptotic cells emit “eat-me” signals that appear on their surface, notifying macrophages that they should be consumed and eliminated. This signaling mechanism directs phagocytic cells, such as macrophages, to engulf and remove the dying cell. “Eat-me” signals include a wide range of chemicals such as nucleotides, phosphatidylserine (PS), and calreticulin (CRT) (15–17). Upon recognition of the dying cell, the phagocyte extends pseudopods to surround and engulf it. This process involves intricate cytoskeletal rearrangements driven by actin polymerization, leading to the formation of phagocytic cups that progressively enclose the dead cell. The engulfment is mediated by various phagocyte receptors, including integrins, scavenger receptors, and complement receptors, which interact with ligands and opsonins on the surface of the dying cell (18–20). After capturing the dying cell, the phagocyte orchestrates a well-coordinated process to ensure its degradation. This begins with the fusion of the phagosome, which contains the dying cell, with lysosomes. Lysosomes are rich in enzymes such as lipases, proteases, and nucleases. As a result of this fusion, the contents of the phagosome are subjected to digestion (11, 21, 22). Following the impairment of efferocytosis, a cascade of events can lead to inflammation. Inflammation occurs when neutrophils, having migrated out of blood vessels to the injured or invaded site, undergo programmed cell death. These neutrophils, along with other inflammatory cells, are then engulfed and cleared by macrophages. This crucial process of phagocytosis plays a vital role in restoring tissue homeostasis as the inflammation gradually subsides (**Figure 1**) (23).

## 3 Ferroptosis and its unique mechanisms

**Figure 2:**
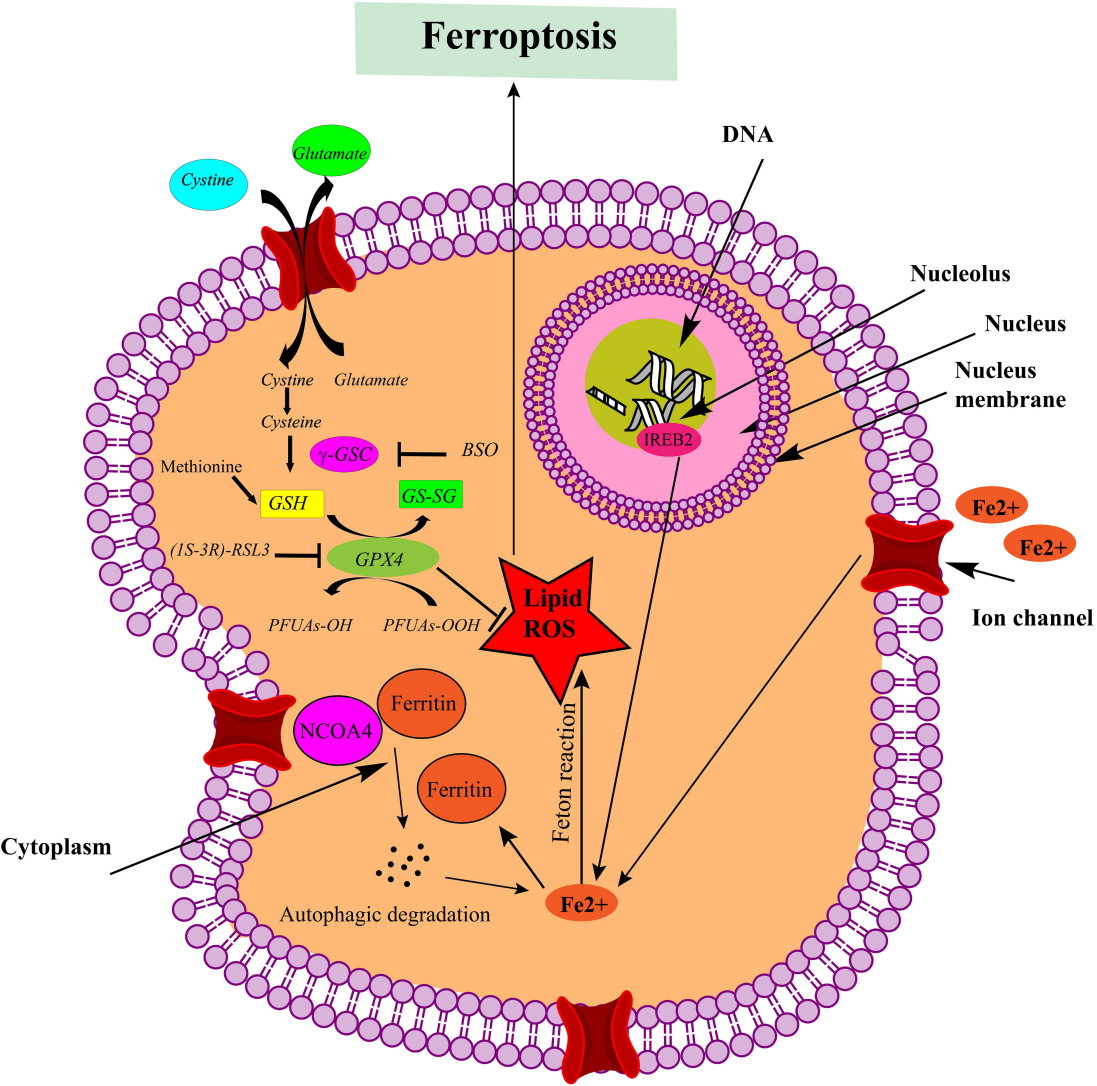
Mechanistic pathways leading to ferroptotic cell death. Ferroptosis is triggered by the inhibition of system Xc- or GPX4 activity, leading to cell death. Lipid ROS are implicated in the ferroptotic process. Notably, peroxidation of polyunsaturated fatty acids (PUFAs) is a significant factor. Excessive iron acts as a fundamental catalyst in the execution of ferroptosis (This figure was created using ChemDraw version 22.2.0.3300, PerkinElmer Informatics.).

Ferroptosis is a specific type of regulated cell death caused by the accumulation of lipid peroxides on cell membranes. It differs from apoptosis and other forms of cell death in terms of both morphology and mechanism. Unlike apoptotic cells, ferroptotic cells do not exhibit classic apoptotic features such as condensed chromatin or the production of apoptotic bodies. Instead, ferroptotic cells are characterized by smaller mitochondria with reduced mitochondrial cristae (24, 25). This process requires a delicate balance between ferroptosis-promoting cellular activities and defense systems that prevent ferroptosis. When ferroptosis-promoting actions surpass the antioxidant-buffering capacities of these defense systems, ferroptosis occurs, leading to cell death (26–29). This distinguishes ferroptosis from other forms of controlled cell death, which generally entail the activation of specific executioner proteins. Examples of such pathways include gasdermin D-mediated pyroptosis, MLKL-mediated necroptosis, and caspase-mediated apoptosis. Ferroptosis, however, follows a distinct pathway, highlighting its unique role in regulated cell death (30). Ferroptotic cells display unique profiles of oxidized phospholipids (PL), which are distinct from those of cells undergoing other types of cell death. This unique composition of oxidized phospholipid further sets ferroptosis apart as a distinct mode of cell demise (31, 32). The mechanistic pathways underlying ferroptotic cell death are illustrated in **Figure 2**, which depicts the key molecular events and signaling cascades involved in the induction and execution of ferroptosis.

## 4 Efferocytosis and ferroptosis in cancer

Efferocytosis and ferroptosis are two separate cellular mechanisms that have garnered significant interest in cancer biology. Although they represent separate processes, there is growing evidence that they play interrelated role in cancer development and progression. Efferocytosis is an important step for maintaining tissue homeostasis and reducing inflammation. In cancer, the deregulation of efferocytosis can have serious consequences. Impaired efferocytosis results in the buildup of apoptotic cells, resulting in persistent inflammation and the release of pro-inflammatory chemicals (33–35). This inflammatory milieu can promote tumor growth, angiogenesis, and metastasis (36–38). Interestingly, recent research highlights the impact of ferroptosis on efferocytic processes in cancer. Dysfunctional iron metabolism and increased lipid peroxidation associated with ferroptosis can hinder efficient efferocytosis, resulting in poor apoptotic cell clearance (39). Impaired clearance can exacerbate inflammation, further contributing to tumor growth. Conversely, apoptotic cells can activate ferroptotic pathways in cancer cells. The release of components from apoptotic cells, such as oxidized lipids and ROS, can induce oxidative stress and ferroptosis in neighboring cancer cells (40–43). The reciprocal interaction between efferocytosis and ferroptosis underscores their interconnected roles within the tumor microenvironment.

## 5 Efferocytosis and ferroptosis in tumor biology

Tumor biology encompasses the study of tumors or neoplasms, which are abnormal growths of cells that can occur in various tissues of the body. Understanding tumor biology is crucial for comprehending the mechanisms underlying cancer development, progression, and potential treatment strategies. Efferocytosis and ferroptosis are two important processes in tumor biology that significantly influence tumor development, progression, and response to therapy. Here’s an overview of their roles in tumor biology:

### 5.1 Efferocytosis in tumor biology

The removal of apoptotic cells by phagocytic cells plays a vital role in tumor biology. This process is essential for preserving tissue homeostasis and regulating the immune response within the tumor microenvironment. Below are some key implications of apoptotic cell clearance in tumor biology:

#### 5.1.1 The impact of efferocytosis on inflammation in the tumor microenvironment

**Figure 3:**
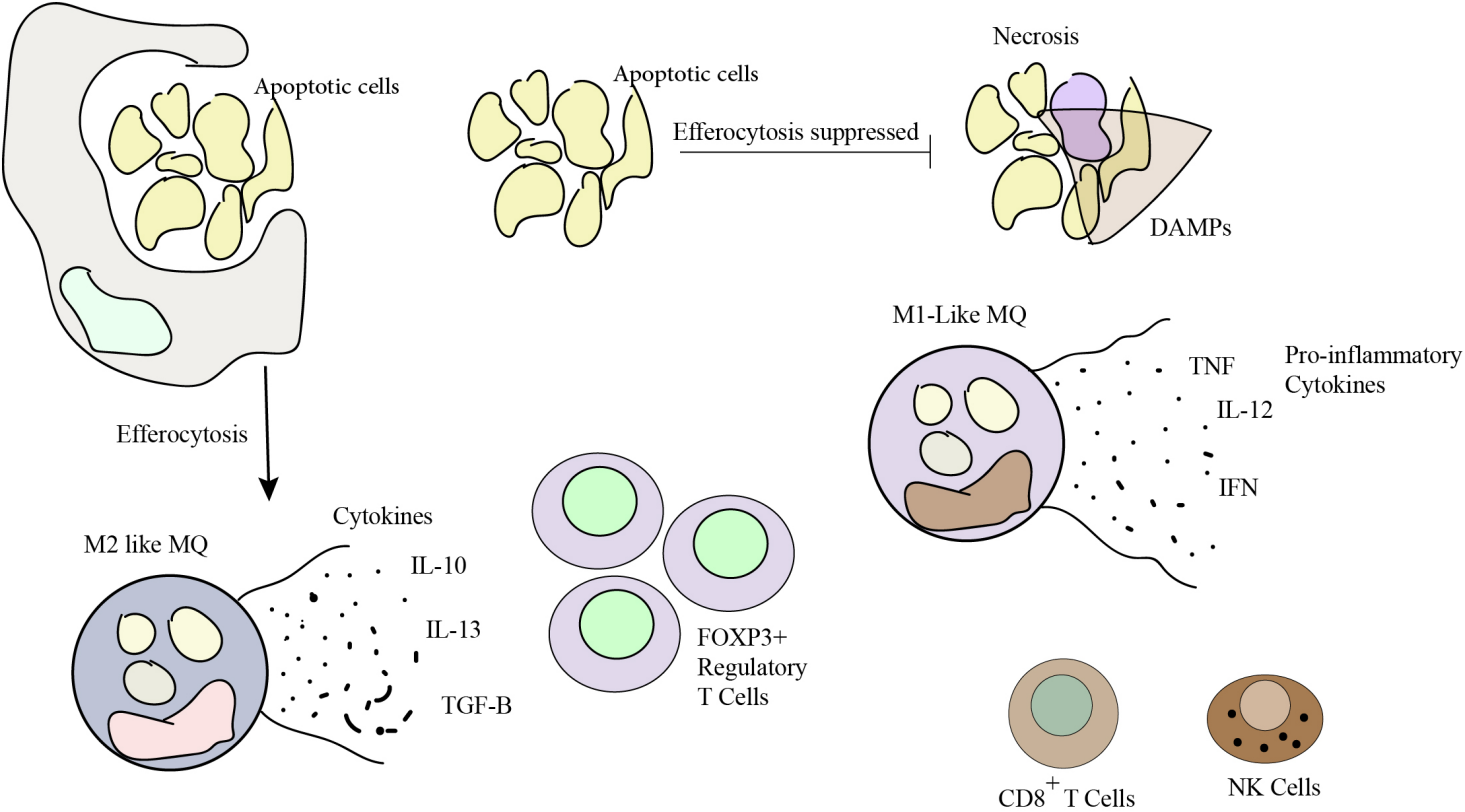
Efferocytosis plays a pivotal role in shaping a pro-tolerogenic tumor microenvironment. A balance in efferocytosis is crucial for sculpting the tumor microenvironment and optimizing immune defenses against cancer. Effective control of efferocytosis holds potential for developing cancer treatments. Efferocytosis that is effective boosts the body’s capacity to fight cancer, while defective efferocytosis causes inflammation and impairs the immune response.

Efficient clearance of apoptotic cells prevents the release of pro-inflammatory molecules and danger signals from dying cells. Failure to clear apoptotic cells can lead to the accumulation of cellular debris, triggering chronic inflammation and activating immune responses. In the context of tumors, persistent inflammation can contribute to tumor progression, angiogenesis, and immune evasion. Proper efferocytosis helps create an immunosuppressive microenvironment and reduces inflammatory responses, thereby influencing tumor growth and metastasis. Additionally, efferocytosis of cancer cells by antigen-presenting cells, such as dendritic cells (DCs), can activate immune responses against tumor antigens. This leads to the priming and activation of tumor-specific T cells, enhancing anti-tumor immunity. Effective efferocytosis promotes the efficient removal of apoptotic cells within the tumor microenvironment, resulting in the polarization of M2-like macrophages, the secretion of wound-healing cytokines (e.g., IL-10, IL-13, TGF-β), and the recruitment of FOXP3+ regulatory T cells. Consequently, a tolerogenic and immunosuppressive tumor microenvironment is established. On the other hand, when efferocytosis is impaired, secondary necrosis occurs, resulting in the release of pro-inflammatory damage-associated molecular patterns (DAMPs). These DAMPs promote the polarization of M1-like macrophages, stimulate the production of pro-inflammatory cytokines (such as TNF, IFN and IL-12), and attract cytotoxic cells, including CD8+ T cells and natural killer cells, which contribute to anti-tumor responses (**Figure 3**) (22, 44–48).

#### 5.1.2 The impact of efferocytosis on tumor antigen release and immune checkpoint expression

Efferocytosis plays a crucial role in recognizing and removing apoptotic tumor cells. Efficient clearance of these dying cells is essential to prevent the release of immunogenic tumor antigens, such as human epidermal growth factor receptor 2 (HER2) and prostate-specific antigen (PSA). These antigens have the potential to trigger an immune response against the tumor (49–52). However, when efferocytosis is impaired, apoptotic tumor cells may not be properly cleared, leading to antigen release and promoting immune activation against the tumor. This defective clearance can have an impact on tumor immune surveillance and influence tumor progression. Moreover, the interaction between phagocytic cells and apoptotic cells also affects immune checkpoint molecules and immune tolerance. When phagocytes engulf apoptotic cells, it can induce the expression of inhibitory immune checkpoint molecules such as PD-L1, LILRB1, TIM3, B7-H3, CD137, and CD47 (53, 54). Increased expression of these immune checkpoints can promote immune tolerance and dampen anti-tumor immune responses (55–57). Thus, the modulation of immune checkpoints through efferocytosis can influence the balance between immune activation and tolerance in the tumor microenvironment.

#### 5.1.3 Metastasis and dormant tumor cells

Efferocytosis of apoptotic tumor cells may impact the metastatic potential of tumors. Efficient clearance of apoptotic cells prevents the accumulation of cellular debris that could stimulate inflammation and promote the recruitment of immune cells involved in metastasis (4, 58, 59). Furthermore, efferocytosis may contribute to the clearance of dormant tumor cells, which can evade immune surveillance and remain latent in distant organs. Impaired efferocytosis can lead to the survival and growth of dormant tumor cells, facilitating metastatic spread. A key factor in the relationship between efferocytosis and metastasis is the origin of macrophages, which influences their localization and function in metastatic environments. Monocyte-derived macrophages (MoMs) and tissue-resident Kupffer cells (KCs) exhibit distinct roles during metastatic progression; while MoMs primarily localize in tumor lesions, KCs are predominantly found at the tumor margins. Studies have revealed that tumor-associated macrophages (TAMs) can exhibit both immunostimulatory and immunosuppressive states within the same tumor (60). Additionally, MoMs accumulate in necrotic regions, where they phagocytose dead resident cells. Inhibiting the phosphatidylserine receptor Mer tyrosine kinase (MerTK) can reverse the immunosuppressive state induced by efferocytosis, as blocking MerTK-mediated phagocytosis of dying cancer cells has been shown to suppress tumor growth in various cancers. In metastatic cancer (61, 62), macrophage accumulation is often observed, accompanied by an immunosuppressive phenotype, that promotes tumor growth and limits the effectiveness of immunotherapy (63, 64). Evidence suggests that both immunostimulatory and immunosuppressive phenotypes of MoMs are present in early and advanced metastatic lesions, with a shift toward immunosuppression occurring at both stages (65). Research by Astuti Y and colleagues indicated that MoMs are constantly attracted to metastatic sites, where they quickly lose their ability to stimulate the immune system. The physiological function of efferocytosis is to shield tissues from immune reactions, which leads to localized immunosuppression. These pathways are used in metastatic cancer to create an immunosuppressive microenvironment, which helps dispersed cancer cells evade immune detection (65).

It has been shown that tissue-resident macrophages are generally more phagocytic than bone marrow-derived macrophages (66). Inhibiting the MerTK can reverse the immunosuppressive state induced by efferocytosis and has been shown to suppress tumor growth in various cancers (46, 67, 68). Notably, MerTK blockade was associated with the accumulation of dead cell debris, activating the STING pathway in macrophages (46).

In summary, Interfering with macrophage efferocytosis or its downstream signaling can inhibit immunosuppressive functions and restore anti-tumor immunity. Targeting macrophage efferocytosis may thus represent a promising therapeutic strategy for patients with metastatic cancer.

### 5.2 Ferroptosis in tumor biology

Ferroptosis plays an important role in tumor biology, encompassing various aspects that contribute to tumor development, progression, and response to therapy. Below are some key implications of ferroptosis in tumor biology:

#### 5.2.1 Tumor suppression

Ferroptosis acts as a tumor-suppressor mechanism by eliminating cancer cells. Unlike normal cells, many cancer cells exhibit altered iron metabolism and increased sensitivity to ferroptosis. Inducing ferroptosis in cancer cells can suppress tumor growth and inhibit their ability to evade cell death (69). By targeting specific vulnerabilities in cancer cells, such as altered lipid metabolism or weakened antioxidant defenses, ferroptosis-inducing agents show promise as potential therapeutic strategies. One of the key factors in the effectiveness of ferroptosis-based therapeutics is the identification of specific vulnerabilities inherent to cancer cells. For instance, many cancer cells have altered lipid metabolism, leading to an accumulation of polyunsaturated fatty acids (PUFAs) that are prone to oxidative damage. Additionally, these cells often have compromised antioxidant systems, which further predisposes them to ferroptosis. Therefore, ferroptosis-inducing agents that exploit these vulnerabilities could offer novel treatment options for cancer patients. Recent studies have also demonstrated that ferroptosis can be triggered by radiotherapy, one of the most widely used cancer treatments (70). Research suggests that ionizing radiation (IR) induces lipid peroxidation—one of the defining features of ferroptosis—through at least two parallel pathways. First, IR generates reactive oxygen species (ROS), which promote lipid peroxidation. This increase in ROS levels can overwhelm the cellular antioxidant defenses, pushing it toward ferroptosis. Second, IR has been shown to upregulate the expression of acyl-CoA synthetase long-chain family member 4 (ACSL4), a key enzyme involved in the biosynthesis of phospholipids enriched with polyunsaturated fatty acids (PUFA-PLs). These specific lipids are particularly susceptible to peroxidation and thereby facilitating the induction of ferroptosis. By combining radiotherapy with ferroptosis-inducing agents, there is potential to enhance therapeutic outcomes by more effectively targeting and eliminating cancer cells (32, 71, 72). However, IR also triggers the expression of ferroptosis inhibitors such as solute carrier family 7 member 11 (SLC7A11) and glutathione peroxidase 4 (GPX4) as part of an adaptive response. The upregulation of SLC7A11, particularly in the context of IR or KEAP1 deficiency, enhances radioresistance by preventing ferroptosis. By inhibiting SLC7A11 or GPX4 with ferroptosis inducers (FINs), radioresistant cancer cells and xenograft tumors can be made more sensitive to IR (70). The tumor-suppressor role of p53 in ferroptosis has been well studied. Gu and colleagues discovered that p53 can enhance ferroptosis by inhibiting the transcription of system xc–subunit SLC7A11, contributing to p53’s tumor-suppressive properties both *in vitro* and *in vivo*. This was observed through a detailed examination of particular lysine acetylation sites on p53 (73, 74). Like p53, BAP1, an epigenetic regulator and tumor suppressor, can also induce ferroptosis by suppressing the expression of SLC7A11 (75). It is unclear, however, how much BAP1’s ferroptosis-promoting activity adds to its tumor-suppressive role, in contrast to p53, whose ferroptosis-promoting activity alone has been proposed to be sufficient to suppress carcinogenesis *in vivo* (74).

#### 5.2.2 Regulation of tumor microenvironment

**Figure 4:**
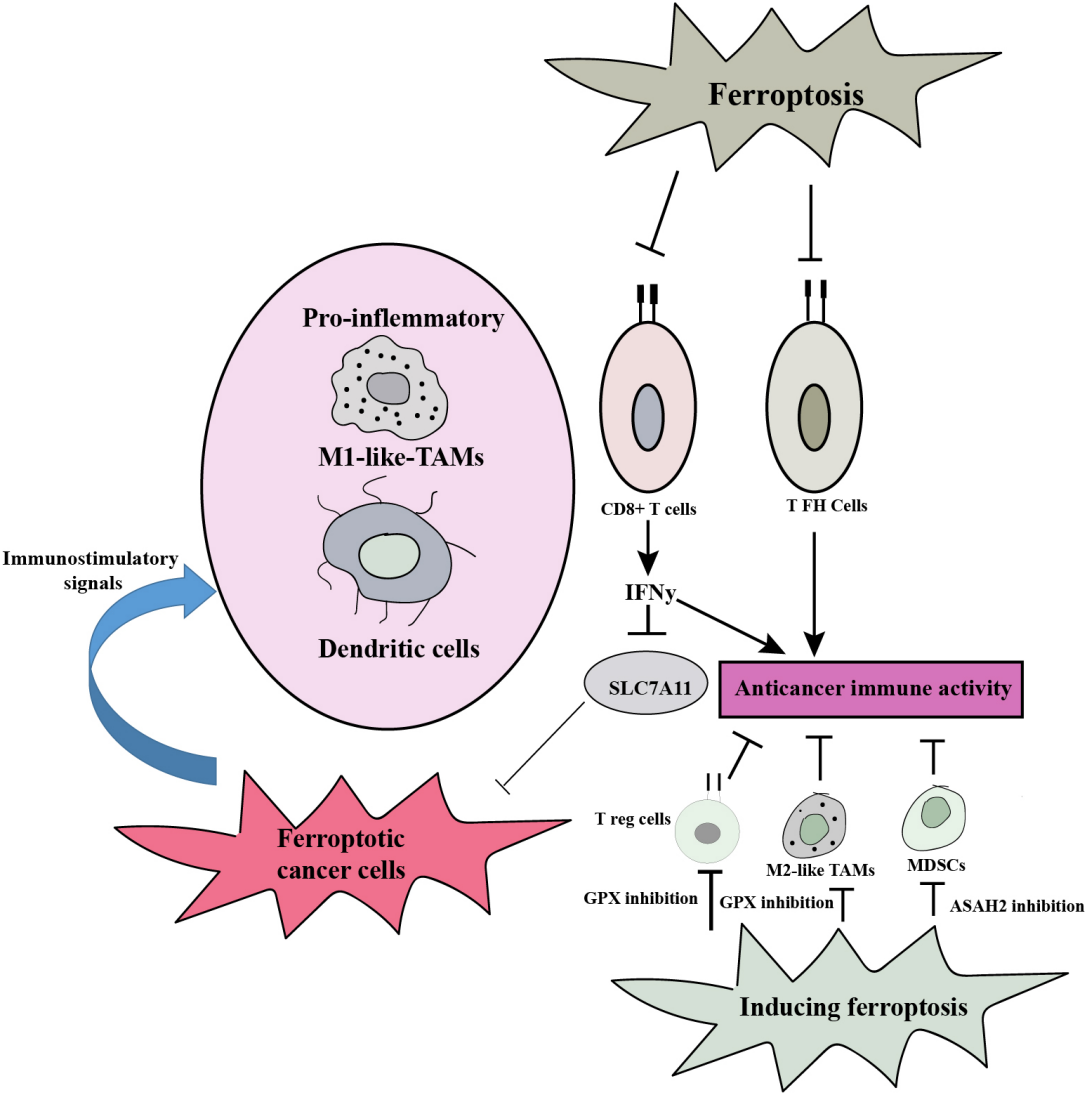
Ferroptosis plays a dual role in antitumor immunity, and the type of immune cell involved determines how it functions. By encouraging ferroptotic cell death in cancer cells and generating immunostimulatory signals that promote DC maturation and M1-like TAM phagocytosis, it can strengthen the immune system’s capacity to fight tumors. By causing cell death in specific immune cell subsets, it can also impair the immunological response. Comprehending these intricate relationships is essential for formulating focused treatment strategies that optimize ferroptosis’ ability to boost antitumor immunity while minimizing any possible negative consequences.

Ferroptosis can influence the tumor microenvironment, which encompasses cellular and non-cellular components surrounding the tumor. Tumor-associated fibroblasts and immune cells can undergo ferroptosis, leading to the release of DAMPs and pro-inflammatory signals. This can shape the immune response, promote antitumor immunity, and modulate tumor progression. Additionally, ferroptosis can affect the availability of nutrients, such as iron, and alter the redox balance within the tumor microenvironment (76). The involvement of ferroptosis in antitumor immunity presents a dual role dependent on the specific immune cell type. The promotion of antitumor immunity is facilitated by CD8+ T cells, which secrete interferon-γ (IFNγ), leading to the repression of SLC7A11 expression in cancer cells and subsequently inducing ferroptosis. Consequently, ferroptotic cancer cells emit immunostimulatory signals that enhance DC maturation and boost the phagocytic ability of macrophages, especially M1-like tumor-associated macrophages (TAMs), enabling efficient clearance of ferroptotic cancer cells. This synergy further strengthens CD8+ T cell-mediated tumor suppression. Furthermore, ferroptosis induction, achieved through the inhibition of GPX4 or N-acyl sphingosine amidohydrolase 2 (ASAH2), impairs various immunosuppressive cell types including regulatory T (Treg) cells, myeloid-derived suppressor cells (MDSCs), and M2-like TAMs. This impairment results in an augmentation of antitumor immunity. However, it is important to note that ferroptosis also affects certain immune cell subsets such as CD8+ T cells and specific T helper (TH) cell subsets like T follicular helper (TFH) cells, compromising the contribution of ferroptosis to antitumor immunity in those cases (**Figure 4**) (29).

#### 5.2.3 Sensitization of resistant tumor cells

Targeting ferroptosis may be a viable strategy to combat drug resistance and improve the therapeutic efficacy of anticancer treatment, according to an increasing body of clinical evidence. The development of resistance in cancer cells to treatments such as lapatinib, cisplatin, docetaxel, sorafenib, and other drugs can be reversed by ferroptosis inducers (77). Conventional chemotherapy or radiation therapy can cause cancer cells to die when xCT and GPX4 are inhibited (78). Through the consumption of glutathione (GSH) and the inhibition of cystine absorption, the inhibition of xCT increases the susceptibility of cancer cells to anticancer drugs (79, 80). It was discovered that via controlling lipid peroxidation, the therapy-resistant high-mesenchymal cell state aids in the escape from ferroptosis. Ferroptosis results from GPX4 inhibition, which triggers peroxide reactions driven by intracellular iron. Therefore, the high-mesenchymal cell state in cancer cells can be successfully eliminated by inducing ferroptosis (81). Some tumor cells develop resistance to conventional cancer therapies, including chemotherapy and targeted therapies. Ferroptosis represents a promising therapeutic pathway for drug-resistant tumor cells and has attracted attention recently. Interestingly, cancer cells that are resistant to apoptosis or other forms of cell death may still retain sensitivity to ferroptosis. This distinctive feature presents an opportunity to exploit ferroptosis as a therapeutic strategy for overcoming resistance to cancer treatment (82, 83). Combining ferroptosis-inducing agents with existing therapies has the potential to sensitize resistant tumor cells, thereby enhancing the overall efficacy of treatment regimens. By targeting the specific metabolic vulnerabilities of these cells, researchers aim to restore their sensitivity to cell death. For instance, the compound erastin has been identified as a potent inducer of ferroptosis. It functions by activating several critical molecular pathways, including system Xc−, which is involved in cystine uptake and glutamate export, VDAC (voltage-dependent anion channel), and p53, a well-known tumor suppressor protein. The activation of these pathways ultimately leads to effective cancer cell death, highlighting the potential of ferroptosis in future therapeutic approaches (84). Despite the promise of erastin, previous studies have shown that a variety of cancer cells exhibit insensitivity to erastin-induced ferroptosis. This insensitivity poses a challenge to the widespread application of ferroptosis in cancer therapy. To address this limitation, researchers have hypothesized that high-dose vitamin C could serve as a potential inducer of ferroptosis in cancer cells. Further experiments have demonstrated that vitamin C can indeed trigger ferroptosis by depleting GSH, and by promoting the generation of ROS. This finding opens new avenues for using vitamin C, not only as a supplement but also as a therapeutic agent in conjunction with ferroptosis-inducing strategies to enhance the treatment of resistant cancer (85).

#### 5.2.4 Tumor dormancy and metastasis

Metastasis presents a significant challenge in cancer treatment, serving as a critical step in tumor progression. Many cancers, such as triple-negative breast cancer (86, 87), cervical cancer (88), and prostate cancer (89, 90), often metastasize, resulting in treatment-related relapses for numerous patients. The differences between primary tumors and their metastatic counterparts are substantial. According to Li et al., there is a lack of effective preventive and therapeutic strategies, greatly affecting patient survival rates (91). Recent research indicates that ferroptosis may play a role in cancer metastasis. This form of regulated cell death could also influence tumor dormancy, as dormant tumor cells enter a non-proliferative state that allows them to resist standard treatments (92). Inducing ferroptosis in dormant tumor cells may prevent their reactivation and subsequent metastatic outgrowth. Additionally, ferroptosis can influence the metastatic potential of tumor cells by modulating their susceptibility to oxidative stress and redox signaling pathways involved in metastasis (92). Several regulators of ferroptosis impact the metastatic capabilities of cancer cells. For instance, NF2, which encodes Neurofibromin 2 (NF2/Merlin), serves as a tumor suppressor that links external signals to intracellular communication, residing in the plasma membrane, cell cortex, and cytoskeleton (93). Another important regulator is linked to the epithelial-mesenchymal transition (EMT), a process by which epithelial cells lose their adhesive properties and acquire a fibroblast-like phenotype, enhancing their migratory and invasive potential. EMT not only facilitates metastasis but also contributes to treatment resistance. Key transcription factors such as ZEB1, SNAI1, and TWIST1 drive these processes and could serve as targets to combat drug resistance and metastasis (8). xCT is another ferroptosis regulator that significantly influences metastasis. Clinical studies have shown notable differences in recurrence rates between xCT-positive and xCT-negative tumors, alongside a correlation between xCT expression levels and metastatic behavior (94). Overexpression of xCT is often associated with poor prognosis in various cancers, including hepatocarcinoma (95, 96). Hypoxia-inducible factor (HIF) is yet another regulator of ferroptosis. In the hypoxic microenvironment characteristic of many tumors, HIF and related genes are activated, promoting cancer cell invasion and metastasis (97, 98). Additionally, noncoding RNAs, such as miR-9 and miR-137, have been shown to regulate ferroptosis during metastatic processes (99). In summary, ferroptosis influences cancer metastasis through various regulators, and understanding these pathways may provide new avenues for therapeutic intervention.

## 6 Exploring the relationship between efferocytosis and ferroptosis

The relationship between efferocytosis and ferroptosis is an intriguing area of investigation that is still being explored. While both processes involve distinct cellular mechanisms, some potential connections and interdependencies have been identified:

### 6.1 Lipid metabolism

Efferocytosis requires dynamic lipid remodeling to facilitate the recognition and engulfment of apoptotic cells (100). This process involves the redistribution of phosphatidylserine (PS) from the inner to the outer leaflet of the apoptotic cell membrane, which serves as an “eat-me” signal for phagocytes (14, 101). Interestingly, alterations in lipid metabolism can also influence the susceptibility of cells to ferroptosis. Certain lipids, such as PUFAs, are prone to peroxidation and can promote ferroptosis (72). Understanding the common lipid pathways and potential regulatory connections between efferocytosis and ferroptosis may provide insights into their relationship.

### 6.2 Oxidative stress

Ferroptosis is characterized by the accumulation of lipid peroxides, resulting from an imbalance between ROS generation and antioxidant defenses (102). Efferocytosis, particularly when impaired or dysregulated, can lead to the accumulation of apoptotic cells and secondary necrosis (103, 104). This can trigger the release of ROS and pro-inflammatory factors, contributing to oxidative stress and potentially influencing the susceptibility of cells to ferroptosis. Conversely, cellular antioxidant systems, which can be regulated by efferocytosis-related pathways, may impact the sensitivity of cells to both oxidative stress and ferroptosis. Lipid hydroperoxides and the depletion of antioxidants like GSH and GPX4 in the metabolism of amino acids are the key ways in which ROS causes ferroptosis. Since macrophages that have a critical role in efferocytosis are the hub of the metabolism of lipids and iron, their associated proteins are very active and involved in the production and elimination of ROS to keep the equilibrium (39).

Furthermore, ROS produced by macrophages initially influences their recruitment and polarization. Typically, the ROS generated within macrophages drive them to polarize towards the M1 phenotype, leading to an increased release of various inflammatory factors that exacerbate inflammation. This process creates conditions conducive to ferroptosis (105, 106). Findings indicate that ROS can have opposing effects on lipid metabolism during ferroptosis. Research revealed that 15-hydroperoxy-eicosa-tetra-enoyl-phosphatidylethanolamine, produced by 15-lipoxygenase (15-LOX), influences the resistance to ferroptosis in M1 macrophages (107). Further studies showed that ROS, such as superoxide (O2) and nitric oxide (NO•), compete for the same entry points and channels leading to the catalytic site of 15-LOX-2 (108). Additionally, because NO• is small enough to pass through cell membranes, it can inhibit phosphatidic acid (PA)-induced ferroptosis in distant epithelial cells (109).

### 6.3 Iron metabolism

Iron, as an essential nutrient, plays a vital role in numerous cellular processes, including DNA synthesis, energy production, and enzymatic reactions. However, excessive or inadequate levels of iron can be detrimental to cellular function and contribute to various pathological conditions. Maintaining the delicate equilibrium of iron homeostasis is therefore of utmost importance (110). Iron is essential for numerous cellular processes, including efferocytosis and ferroptosis. Efferocytosis requires iron for the proper functioning of phagocytes involved in engulfing apoptotic cells (111). Iron can affect the fate and function of effector macrophages in efferocytosis, particularly in relation to cell proliferation and differentiation. Macrophages frequently bind iron to ferritin (Ft). The expression of genes related to iron will change based on the macrophage polarization stage (112). Research indicates that iron overload can promote M1 macrophage polarization by elevating levels of M1 markers like IL-6, TNF-α, and IL-1β and lowering levels of M2 producers like TGM2 (113).

Dysregulated iron metabolism is also associated with ferroptosis, as iron can participate in the Fenton reaction, leading to ROS production and lipid peroxidation (114–116). Disruption in iron metabolism can disturb the delicate balance between efferocytosis and ferroptosis, resulting in pathological consequences (69, 111, 117). Understanding how iron levels influence efferocytosis efficiency and susceptibility to ferroptosis can aid in the development of new therapy targets and approaches for a wide range of diseases, including cancer, neurological problems, and cardiovascular maladies. Furthermore, it could lead to novel therapeutic approaches aimed at restoring cellular equilibrium and improving health.

### 6.4 Inflammatory signaling

Chronic inflammation is associated with various diseases, including cancer (118–120). Efferocytosis can influence the resolution of inflammation by promoting the clearance of apoptotic cells and the release of anti-inflammatory signals (121). Conversely, ferroptosis can lead to the release of pro-inflammatory molecules such as IL-6, iNOS, TNF-α, IL-1β, and COX-2) (122, 123). Interestingly, specific cytokines produced by macrophages can both promote and inhibit ferroptosis. For instance, IL-6, a marker associated with M1 macrophages, enhances lipid peroxidation and disrupts iron homeostasis in bronchial epithelial cells, thereby facilitating ferroptosis (124, 125). It does so by regulating hepcidin, a peptide hormone that affects iron absorption and mobilization in macrophages through the JAK-STAT3 pathway, while also being influenced by the BMP/SMAD signaling pathway (126, 127). TNF-α contributes to ferroptosis by upregulating ACSL3, an enzyme involved in acyl-CoA synthesis, which promotes lipid accumulation and fosters an inflammatory environment conducive to ferroptosis (128). IL-1β can increase the expression of ferroportin (FPN) via the p38-MAPK pathway, potentially leading to excessive iron efflux and accumulation in surrounding nerve cells (129). Furthermore, IL-1β released by macrophages has been shown to upregulate hepcidin transcription by improving the expression of CCAAT enhancer-binding protein (C/EBP) (130, 131) and hepcidin expression through phosphorylated c-Jun N-terminal kinase and its substrates, c-Jun and JunB, ultimately resulting in iron overload and FPN degradation (132). iNOS, although primarily known as a pro-inflammatory factor, has a complex role in ferroptosis. While its activation can produce reactive oxygen and nitrogen species that deplete glutathione and exacerbate lipid peroxidation, studies suggest that inhibiting iNOS may worsen conditions in certain contexts, such as beta-cell death, by promoting lipid peroxidation in the presence of inflammatory cytokines (133–135).

Efficient efferocytosis can limit the release of ROS and lipid peroxides from apoptotic cells, thereby reducing the induction of ferroptosis in neighboring cells. Additionally, apoptotic cells can influence the expression of key molecules involved in ferroptosis, including iron transporters and lipid metabolism enzymes. This modulation potentially sensitizes adjacent cells to ferroptosis (136–140). These findings highlight the intricate interplay and regulatory mechanisms between efferocytosis and ferroptosis, unveiling their impact on cellular responses and fate. Investigating the cross-regulation between inflammatory signaling pathways and the interplay between efferocytosis and ferroptosis could provide important insights into disease pathogenesis.

### 6.5 Therapeutic implications

The interplay between efferocytosis and ferroptosis has therapeutic implications. Modulating efferocytosis can potentially regulate ferroptosis and vice versa. Enhancing efferocytosis or inhibiting ferroptosis may have therapeutic benefits in certain disease contexts. For example, in cancer, promoting efferocytosis can help clear apoptotic cancer cells and suppress inflammation, while inhibiting ferroptosis can protect normal tissues from oxidative damage (141, 142).

Unraveling the intricate link between efferocytosis and ferroptosis holds promise for understanding the underlying mechanisms of various diseases and exploring novel therapeutic avenues. Future research in this area will likely uncover additional regulatory mechanisms and therapeutic targets, offering opportunities for interventions aimed at restoring tissue homeostasis and mitigating disease progression. The main properties of ferroptosis and efferocytosis are shown in **Table 1**.

**Table 1:** The primary characteristics of ferroptosis and efferocytosis.

Main characteristics	Efferocytosis	Ferroptosis
**Biochemical characteristics**	Recognition of apoptotic cells by phagocytes, activation of signaling pathways, cytoskeletal rearrangements, and engulfment and internalization of apoptotic cells	Lipid peroxidation, iron accumulation, depletion of glutathione and GPX4, and dysregulation of redox homeostasis
**Morphological characteristics**	Formation of phagosomes or phagolysosomes	reduced cristae density, increased membrane density, presence of condensed mitochondria, cytoplasmic and nuclear shrinkage, increased membrane blebbing, absence of chromatin condensation or nuclear fragmentation
**Key genes**	MERTK, AXL, TYRO3, TIM-4, CD36, and integrins such as αvβ3 and αvβ5	GPX4, ACSL4, FSP1 (ferroptosis suppressor protein 1), and SLC7A11
**Regulatory pathways**	The MerTK, AXL, and TYRO3 receptor tyrosine kinase pathways, as well as the phosphatidylserine signaling pathway	HSF1-HSPB1, xCT and Gpx4, p62-Keap1-Nrf2 pathway, MVA, LSH signal pathway
**Immune characteristics**	Mostly anti-inflammatory (through the release of IL-10 and TGF-β, the promotion of immune tolerance through the induction of Tregs)	Pro-inflammatory (the release of DAMPs, the recruitment, and activation of immune cells, the potential modulation of tumor microenvironment, and anti-tumor immune responses)
**Inducers**	Phosphatidylserine exposure, calreticulin exposure, and nucleotides like ATP, MFG-E8, and Gas6	Erastin, FIN56, sorafenib, and artesunate, DPI7, DPI10, GPX4 inhibitors, such as RSL3 and ML162
**Inhibitors**	Annexin V, blocking antibodies against efferocytosis receptors like MERTK, AXL, and TIM-4, interferes with the factor of cytochalasin D and actin filaments	Ferrostatins (ferrostatin-1, ferrostatin-4), liproxstatins, and iron chelators like deferoxamine, antioxidants, and lipophilic radical scavengers

## 7 Implications of dysregulated cell clearance and cell death in cancer

Dysregulated cell clearance and cell death processes have profound implications for cancer development and progression (143, 144). The inability to properly clear dying or damaged cells and the dysregulation of cell death pathways can disrupt tissue homeostasis and contribute to various aspects of tumor biology. Dysregulated cell clearance and cell death can lead to the accumulation of apoptotic cells, necrotic debris, and cellular waste in the tumor microenvironment. This sets off an inflammatory reaction that manifests as the release of DAMPs, chemokines, and pro-inflammatory cytokines (145). These molecules act as danger signals, alerting the immune system to tissue damage and cellular stress. Chronic inflammation creates an environment that promotes tumor growth, angiogenesis, and metastasis, providing a favorable niche for cancer cells to thrive (146). Impaired clearance of dying cells hinders the immune system’s capacity to recognize and eliminate cancer cells effectively. This dysfunction not only reduces the immune response to tumors but also fosters an immune-tolerant environment that can further facilitate cancer progression (3, 147). The release of self-antigens from uncleared apoptotic cells can trigger autoimmunity, while the accumulation of apoptotic cells can induce immune tolerance, providing a survival advantage to cancer cells (148–150). Understanding the intricate relationship between impaired cell clearance, immune dysregulation, and cancer progression is crucial for the development of therapeutic strategies aimed at enhancing efferocytosis and restoring immune surveillance to combat cancer effectively. Dysregulated cell clearance and cell death contribute to tumor progression and metastatic spread. Inefficient clearance of apoptotic cells can promote the survival and proliferation of cancer cells, leading to the expansion of the tumor mass. Moreover, the accumulation of necrotic debris and DAMPs can stimulate tumor cell invasion, angiogenesis, and the remodeling of the extracellular matrix, facilitating tumor metastasis (151).

Aberrant cell clearance and modified cell death mechanisms can confer resistance to cancer therapies (152–154). Cancer cells that evade apoptosis or undergo alternative cell death modalities such as autophagy or necrosis may survive treatment interventions. Additionally, impaired clearance of dying cells can lead to the persistence of therapy-induced cellular debris, triggering inflammation and promoting treatment resistance (155).

Dysregulated cell death and impaired clearance can contribute to genomic instability, a hallmark of cancer. Incomplete or defective cell death processes can result in the accumulation of damaged DNA and genomic alterations (156). Genomic instability provides a fertile ground for the acquisition of oncogenic mutations, driving tumor progression and therapeutic resistance.

## 8 Manipulating efferocytosis and ferroptosis for improved cancer therapy

Manipulating efferocytosis and ferroptosis to improve cancer therapy involves employing various strategies to enhance or inhibit these processes selectively. Here are some approaches being explored to leverage efferocytosis and ferroptosis for improved cancer treatment:

### 8.1 Enhancing efferocytosis

Promoting efficient clearance of apoptotic cells by enhancing efferocytosis can reduce inflammation and prevent secondary necrosis, which is associated with tumor progression. This can be achieved by targeting signaling pathways involved in efferocytosis, such as the MerTK receptor or the phosphatidylserine (PS) recognition pathway (157–160).

#### 8.1.1 Phagocytic receptor agonists

Phagocytic receptor agonists are compounds that stimulate immune cells, including macrophages and neutrophils, by attaching to particular receptors on their surfaces. These receptors are crucial for identifying and ingesting harmful pathogens, dead cells, and other cellular waste. Among the various types of phagocytic receptors for apoptotic cells are TIM-1, TIM-4, Stabilin-2, and BAI-1, which interact with phosphatidylserine, a molecule present on the surface of dying cells (161–163). Other important receptors include Lactadherin, αVβ3, αVβ5, CD36, and CD14. Notably, Lactadherin and αVβ3 also bind to milk fat globule-epidermal growth factor 8 (MFG-E8) (164), while oxidized lipids act as ligands for CD36 (165). Examples of phagocytic receptor agonists include laminarin, molecules with terminal mannose, and N-Formyl-methioninyl-leucyl-phenylalanine (F-MLF) (166). Terminal mannose is specifically recognized by the mannose receptor, primarily located on macrophages (167). F-MLF activates formylmethionine phagocytic receptors (FPRs), with seven types identified in mice and three in humans (168). Additionally, various synthetic compounds have been developed to activate specific phagocytic receptors. These agonists promote receptor clustering and activate signaling pathways that lead to cytoskeletal rearrangement, enhancing the uptake of apoptotic cells. Moreover, stimulating phagocytic receptors often triggers the release of pro-inflammatory cytokines, further activating the immune response. They can also improve T cell activation by enhancing antigen presentation by phagocytes (169).

Activation of phagocytic receptors, such as MerTK or CD47-SIRPα, can enhance efferocytosis. Agonistic antibodies or small molecules targeting these receptors have been investigated to stimulate phagocytosis of apoptotic cancer cells and promote tumor clearance. In non-small lung cell carcinoma, gastric cancer cells, head and neck cancers, and glioblastoma, there have been observed advantages in targeting the reduction or inhibition of MerTK activity (170, 171).

#### 8.1.2 Lipid mediators

Lipid mediators, such as specialized pro-resolving mediators (SPMs) like resolvins and protectins, can promote efferocytosis and dampen inflammation. Administration of exogenous SPMs or modulation of endogenous SPM production has shown the potential to enhance efferocytosis and limit tumor progression. It was shown that SPMs play a crucial role in actively resolving inflammation. However, when inflammation remains unresolved, chronic inflammation creates a favorable environment that promotes carcinogenesis and cancer progression (172). Conventional cancer therapies can further enhance cancer-related inflammation by inducing extensive tumor cell death, leading to the activation of immune-infiltrating cells such as TAMs (173, 174). Macrophages, with their versatile phenotype, are central players in both inflammation and its active resolution. Exploiting their high plasticity, cancer cells can manipulate macrophages to support tumor progression, immune evasion, and therapy resistance. The impaired resolution of cancer-associated inflammation, mediated by TAMs, can thus reinforce tumor progression. Excitingly, recent evidence suggests that stimulating the pro-resolving functions of macrophages using SPMs can promote inflammation resolution in cancer and enhance the effectiveness of anticancer treatments (175).

#### 8.1.3 Immune checkpoint blockade

Immune checkpoint inhibitors have emerged as promising therapeutic agents in cancer treatment. Specifically, antibodies targeting programmed cell death protein 1 (PD-1) or programmed death-ligand 1 (PD-L1) have demonstrated the potential to not only restore immune responses but also exert a positive influence on efferocytosis. By blocking the PD-1/PD-L1 interaction, these inhibitors effectively inhibit the inhibitory signals that suppress immune cell activity. One of the notable effects of immune checkpoint inhibitors is their ability to enhance the phagocytic activity of immune cells involved in efferocytosis. These inhibitors promote the recognition and clearance of apoptotic cells by immune cells, such as macrophages and dendritic cells, through the upregulation of phagocytic receptors and the improvement of their functional capacity. Consequently, the efficient clearance of apoptotic cells is facilitated, preventing the accumulation of dead cells and the subsequent release of pro-inflammatory molecules. Furthermore, immune checkpoint inhibitors play a crucial role in reinvigorating immune responses against cancer cells. By blocking the PD-1/PD-L1 interaction, they unleash the cytotoxic potential of immune cells, such as T cells, enabling them to effectively target and eliminate tumor cells (176). This enhanced immune response contributes to a more favorable tumor microenvironment, favoring efferocytosis and reducing inflammation. Overall, the administration of immune checkpoint inhibitors, specifically antibodies targeting PD-1 or PD-L1, holds promise in not only restoring immune responses but also promoting efferocytosis and reducing inflammation. The combined effects of immune checkpoint inhibitors on immune cell activity and their ability to restore the balance between immune tolerance and effective tumor recognition make them a valuable approach in cancer therapy (46).

#### 8.1.4 Synergistic approaches

Combining therapies that induce apoptosis, such as chemotherapy or targeted therapies, with agents that enhance efferocytosis can improve treatment response. The clearance of apoptotic cancer cells reduces the release of pro-inflammatory factors and potentially enhances the anti-tumor immune response.

### 8.2 Inducing ferroptosis

#### 8.2.1 Lipid peroxidation inducers

Small molecules that induce lipid peroxidation and ferroptosis, such as erastin and RSL3, have garnered significant attention as potential anti-cancer agents. These compounds offer a targeted approach to selectively trigger ferroptosis in cancer cells, leading to their demise, while sparing normal cells from excessive damage and toxicity (177, 178). Erastin, for instance, has been extensively studied for its ability to selectively induce ferroptosis in cancer cells. It functions by inhibiting the activity of system xc-, a cystine/glutamate antiporter that plays a crucial role in importing cystine into cells in exchange for glutamate. By blocking system xc-, erastin disrupts the cellular supply of cystine, thereby impairing the synthesis of glutathione, the key antioxidant molecule that protects cells from oxidative stress. Consequently, cancer cells with high metabolic demands and increased dependency on cystine uptake become particularly vulnerable to erastin-induced ferroptosis (179). Similarly, RSL3 acts as a potent inducer of ferroptosis by specifically targeting and inhibiting the activity of GPX4. GPX4 plays a critical role in neutralizing lipid peroxides and suppressing ferroptosis. By inhibiting GPX4, RSL3 disrupts the cellular defense mechanisms against lipid peroxidation, leading to the accumulation of toxic lipid peroxides and subsequent initiation of ferroptosis in cancer cells (180).

#### 8.2.2 Glutathione depletion

Ferroptosis relies on the depletion of intracellular antioxidant systems, primarily glutathione (181, 182). Glutathione acts as a critical line of defense against oxidative stress by neutralizing reactive oxygen species and inhibiting lipid peroxidation, thus preventing ferroptosis. However, inhibiting glutathione synthesis can sensitize cancer cells to ferroptosis-inducing agents and significantly enhance treatment efficacy (183). One approach to deplete intracellular glutathione levels and promote ferroptosis is the use of inhibitors targeting glutathione synthesis. Buthionine sulfoximine (BSO) is a potent inhibitor of γ-glutamylcysteine synthetase, the rate-limiting enzyme in glutathione biosynthesis. By inhibiting this enzyme, BSO impairs the synthesis of glutathione, leading to a reduction in intracellular glutathione levels. Consequently, the decreased availability of glutathione renders cancer cells more susceptible to ferroptosis induction (184, 185). When combined with ferroptosis-inducing agents, such as small molecules that inhibit the activity of GPX4, BSO can significantly potentiate the therapeutic effects. GPX4 is a critical enzyme that suppresses lipid peroxidation and functions as a key regulator of ferroptosis. Inhibition of GPX4 allows the accumulation of lipid peroxides, ultimately leading to the initiation of ferroptosis. By inhibiting glutathione synthesis, BSO disrupts the cellular antioxidant defense system, rendering cancer cells more vulnerable to the accumulation of lipid peroxides and subsequent ferroptotic cell death (186).

#### 8.2.3 Iron-dependent ferroptosis

**Figure 5:**
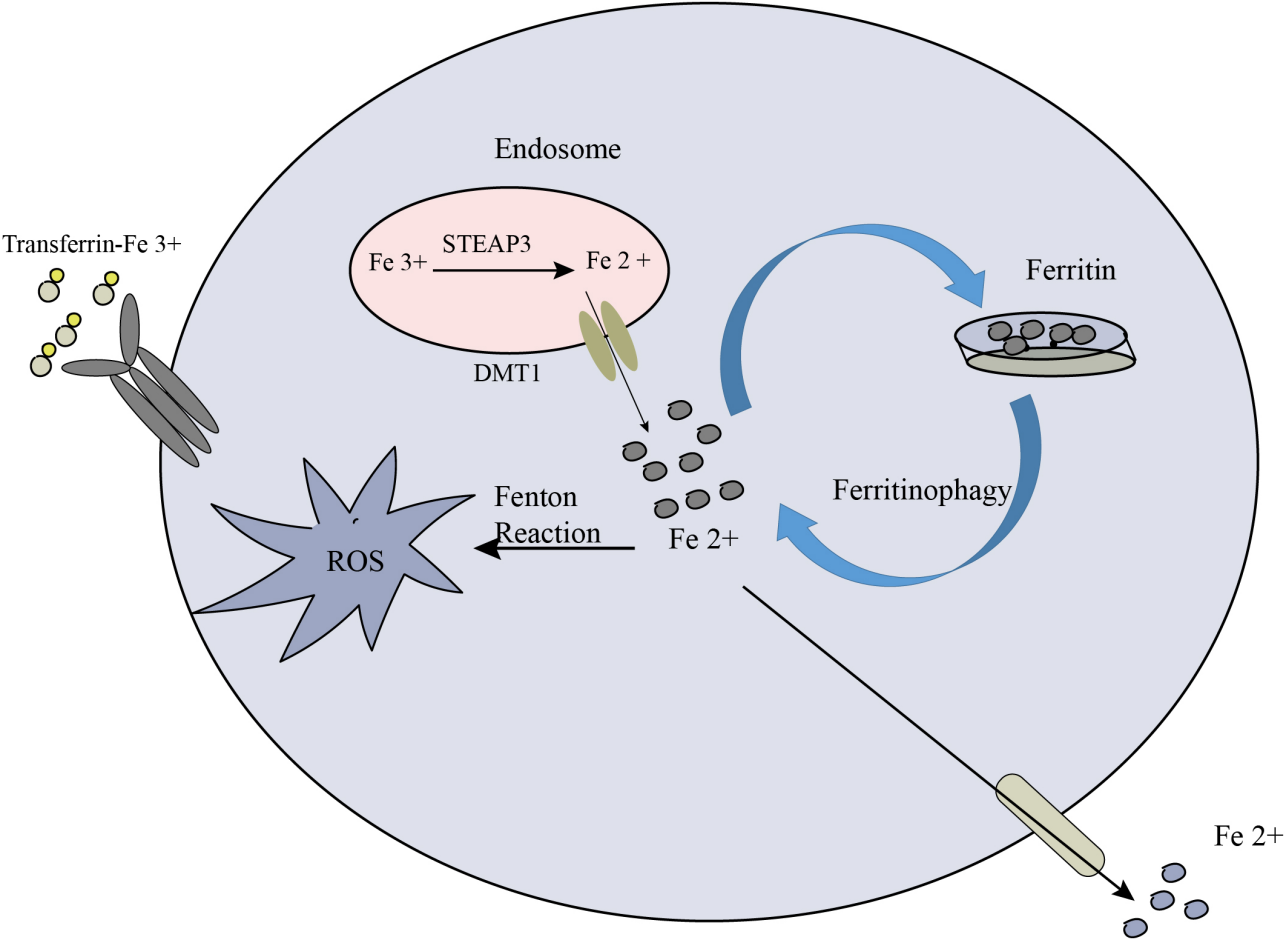
The crucial role of iron metabolism in the process of ferroptosis. Cellular iron homeostasis relies on the coordinated regulation of iron uptake, export, utilization, and storage.

Modulating iron metabolism and availability is a key strategy to influence ferroptosis. By targeting iron homeostasis, it is possible to regulate intracellular iron levels and subsequently impact the potential for lipid peroxidation and ferroptosis. This approach offers a promising avenue for anti-cancer therapy (187). One approach to modulating iron metabolism is through the use of iron chelators or iron transport inhibitors. Iron chelators such as deferoxamine and deferiprone are compounds that bind to iron ions, forming stable complexes and reducing the availability of free iron within cells. By sequestering iron, chelators limit its participation in Fenton reactions, which generate harmful reactive oxygen species that can trigger lipid peroxidation. Consequently, iron chelators can reduce the potential for lipid peroxidation and, subsequently, ferroptosis induction. In addition to iron chelation, inhibiting iron transporters or channels involved in iron uptake can also limit intracellular iron levels. For instance, inhibiting transferrin receptor 1 (TfR1), which facilitates iron uptake via transferrin-bound iron, can restrict iron availability for cellular processes. This, in turn, can reduce the availability of iron for Fenton reactions and lipid peroxidation, thereby modulating the susceptibility of cancer cells to ferroptosis. In the extracellular environment, Fe3+ binds to transferrin and is internalized into cells via TFR1. Within the endosome, STEAP3 (six-transmembrane epithelial antigen of prostate 3) metalloreductases facilitate the reduction of Fe3+ to Fe2+. Subsequently, divalent metal transporter 1 (DMT1) transports Fe2+ from the endosome to the cytoplasm, where it enters a labile iron pool. To maintain plasma iron levels, the labile iron is exported through the membrane protein ferroportin. Alternatively, excess iron from the labile iron pool can be sequestered in ferritin heteropolymers (such as ferritin heavy chain 1 or ferritin light chain), which represents a redox-inactive form of iron. This storage mechanism serves to safeguard cells and tissues from iron-induced damage. Notably, ferritinophagy, the autophagic degradation of ferritin, contributes to ferroptosis by elevating labile iron levels. The process involves the participation of autophagy-related proteins (Atg5 and Atg7) and nuclear receptor coactivator 4 (NCOA4) (188). Overall, the coordinated regulation of iron metabolism and its impact on ferroptosis are depicted in **Figure 5**.

#### 8.2.4 Salinomycin-loaded gold nanoparticles

Gold nanoparticles functionalized with salinomycin have been utilized as a targeted delivery system to induce ferroptosis in cancer cells (189). Salinomycin is a compound known to disrupt cellular processes related to iron metabolism and GSH homeostasis. By encapsulating salinomycin within gold nanoparticles, the drug can be specifically delivered to cancer cells. Once internalized, salinomycin-loaded AuNPs can induce iron accumulation within the cells, leading to increased levels of labile iron. This iron overload disrupts redox balance and exhausts intracellular GSH, a critical component of the cellular antioxidant defense system. As a result, cancer cells become highly susceptible to oxidative damage and ferroptosis (190).

#### 8.2.5 Lipid peroxidation generator with a GSH and iron redox couple

Another approach to induce ferroptosis involves the use of a novel redox couple composed of GSH and iron. GSH, a key intracellular antioxidant, can undergo redox reactions with iron to generate ROS and initiate lipid peroxidation. By manipulating this redox couple, researchers have developed compounds that promote the production of ROS and subsequent lipid peroxidation. This process overwhelms the cellular antioxidant defenses and triggers ferroptosis in cancer cells. This strategy provides a targeted and selective means to induce ferroptosis, exploiting the vulnerabilities of cancer cells with dysregulated iron metabolism and antioxidant systems (191).

#### 8.2.6 Oxygen-boosted phototherapy

Phototherapy techniques, such as photodynamic therapy (PDT), have been combined with strategies to boost oxygen levels within cancer cells. PDT involves the use of light-sensitive compounds, known as photosensitizers, which generate reactive oxygen species upon exposure to specific wavelengths of light. By enhancing the availability of oxygen in the tumor microenvironment, either through oxygen delivery or modulation of hypoxia, the effectiveness of PDT in inducing ferroptosis can be amplified. Increased oxygen levels facilitate the generation of ROS, leading to lipid peroxidation and ferroptotic cell death in cancer cells (192, 193).

### 8.3 Combination approaches

#### 8.3.1 Efferocytosis-ferroptosis interplay

Synergistic effects may be achieved by combining strategies that enhance efferocytosis with agents that induce ferroptosis. Efficient clearance of apoptotic cancer cells can reduce inflammation and create a microenvironment favorable for ferroptosis induction, leading to enhanced tumor cell death.

#### 8.3.2 Concurrent treatments

Integrating efferocytosis and ferroptosis-targeting approaches with standard treatments, such as chemotherapy, radiation therapy, or immunotherapy, can improve treatment response and overcome resistance mechanisms. Customized combination regimens based on the specific characteristics of the tumor and the patient may optimize therapeutic outcomes.

It is important to note that the manipulation of efferocytosis and ferroptosis in cancer therapy is still an area of active research, and further studies are needed to optimize treatment strategies, understand potential side effects, and identify biomarkers for patient selection. Nonetheless, these approaches offer promising avenues for developing innovative and more effective cancer therapeutics that exploit the intricate interplay between efferocytosis and ferroptosis.

## 9 Conclusion

The interplay between efferocytosis and ferroptosis has attracted significant interest in cancer research. Efferocytosis is crucial for maintaining tissue balance, while dysregulation can lead to chronic inflammation. Dysregulated ferroptosis, on the other hand, can hinder efficient efferocytosis and impede apoptotic cell clearance. Conversely, apoptotic cells can activate ferroptotic pathways, inducing cell death in cancer cells. Understanding and managing the delicate balance between efferocytosis and ferroptosis could have therapeutic implications in cancer treatment. Targeting these pathways may improve tumor clearance, reduce inflammation, and enhance the effectiveness of existing therapies. Further research in this area is necessary to develop novel therapeutic approaches that restore tissue homeostasis and improve patient outcomes.

## Glossary

ACs: Apoptotic cells
ACSL4: Acyl-CoA synthetase long-chain family member 4
CRT: Calreticulin
DAMPs: Damage-associated molecular patterns
DMT1: Divalent metal transporter 1
FOXP3: Forkhead box P3
GPX4: Glutathione peroxidase 4
HER2: Human epidermal growth factor receptor 2
HSF1: Heat shock factor-1
HSPB1: Heat shock protein beta-1
IFN: Interferon
IL: Interleukin
Keap1: Keleh-like ECH-associated protein 1
LILRB1: Leukocyte immunoglobulin-like receptor subfamily B member 1
LSH: Lymphoid-specific helicase
MVA: Mevalonate
NCOA4: Nuclear receptor coactivator 4
Nrf2: Nuclear factor erythroid 2-related factor 2
PD-L1: Programmed death-ligand 1
PL: Phospholipids
PS: Phosphatidylserine
PSA: Prostate-specific antigen
PUFAs: polyunsaturated fatty acids
ROS: Reactive oxygen species
RSL3: Ras-selective lethal small molecule 3
SLC7A11: Solute carrier family 7 member 11
STEAP3: Six-transmembrane epithelial antigen of the prostate 3
TFR1: Transferrin receptor 1
TGF-β: Transforming growth factor beta
TIM3: T-cell immunoglobulin and mucin domain- containing protein 3
TNF: Tumor necrosis factor
xCT: System xc-
